# Optimization study on spatial distribution of rice based on a virtual plant approach

**DOI:** 10.1371/journal.pone.0243717

**Published:** 2020-12-17

**Authors:** Lifeng Xu, Zusheng Huang, Zhongzhu Yang, Weilong Ding, Gerhard Hartwig Buck-Sorlin

**Affiliations:** 1 College of Computer Science & Technology, Zhejiang University of Technology, Hangzhou, P.R. China; 2 Institut Agro, University Angers, Angers, France; Universiti Sains Malaysia, MALAYSIA

## Abstract

How to increase crop yield is the most important issue in agricultural production. Many studies have been devoted to optimizing spatial distribution of crops, to improve light interception and increase photosynthetic assimilation. However, finding an optimal solution based on field experiments is almost impossible since the large number of combinations of factors that are related, and the cost in terms of finances and time are prohibitive. A new optimization strategy was proposed in this study, integrating a Functional-Structural Model of rice with a workflow based on a Mixed Particle Swarm Optimization (MPSO) algorithm. The 3D modelling platform GroIMP was used to implement the model and optimization workflow. MPSO is a new Particle Swarm Optimization-based algorithm with multistage disturbances, which has improved abilities to get rid of local optima and to explore solution space. Spacing between plants was used as optimization target in the first example. An optimal plant spacing was obtained within the model framework of current environmental settings together with the functional and structural modules. Simulation results indicate that the optimized plant spacing could increase rice yield, and that the optimization results remain stable.

## Introduction

Crop yields need to be increased due to the current shortage of food, especially cereals, which serves as staple foods for an incessantly increasing human population. Unlike crop breeding, which takes several years if not decades, the optimization of crop type, plant density and planting pattern is a method that can be made available at once. Plant spacing has a great influence on the growth of plants: a very high density can lead to intense competition for nutrients in the soil between neighbouring crops, while excessive spacing is a waste of planting area. An appropriate plant spacing is needed, but it is very expensive in time, financial resources and material power to do research in real life on this question, and the results will in any case always be connected with the specific growing environment at the time of the experiment, thereby rendering generalizations of results difficult. Recent research [1] in 3D plant modelling has well demonstrated that the essential elements of growth mechanisms and physiological processes can be integrated in virtual crop plants, which means that the virtual plant model could well be an appropriate placeholder for the real plant when doing optimization research. FSPM or Virtual Plant Modelling is a paradigm that is considering not only static plant architecture (as a snapshot of plant morphology at a certain developmental stage), but incorporating patterns of organ formation by defining organs/botanical entities as modules, which allows a fully object-oriented approach to programming. Multi-scaled 3D models are increasingly used to characterize plant structure and function. A 3D rice model [2] combined data from quantitative genetics, morphology, and crop physiology in a comprehensive genotype-phenotype modelling framework, i.e. the reconstruction of rice morphology from growth rules, QTL-genotype modifying model parameters, and interaction with a simulated light environment. This framework also allowed some basic “virtual breeding” [3].

Mixed Particle Swarm Optimization (MPSO, unpublished), which has been developed based on PSO [4], was used for optimizing plant spacing in a rice model because of its excellent exploration and mining capabilities. In this paper, MPSO was used to tackle the problem of finding an optimal plant spacing in rice with respect to light interception and resulting (grain) biomass production; the plant spacing was used as a variable in a rice model population to explore changes in the (grain) biomass of model.

The majority of model-based optimization approaches for planting schemes is based on actual crop plants. Ehsanullah *et al*. performed an experiment on the effects of different sowing methods on plant populations and yield of rice (*Oryza sativa* L., cv ‘Super Basmati’) in Pakistan [5], and the results showed that transplanting at 20 cm row distance proved to be the most productive planting density for the cultivation of this cultivar. Based on a given ratio of 2:1 in maize and soybean intercropping, Zhu *et al*. investigated the detailed plant configuration [6]. They found that the optimized plant configuration consisted in a row distance of 25 cm and an inter-plant distance of 25 cm in the case of maize plants, furthermore 30 cm row distance between soybean and maize, and 10 cm between soybean plants. Wang *et al*. carried out a field experiment to provide the theoretical basis [7] for the study of the effects of different intercropping planting patterns on growth and quality of cotton: they found out that line spacing between cotton and date plants impacted mainly the process of boll-splitting. Luo *et al*. [8] used biogeography based on a Gaussian distribution to solve a tomato planting planning problem by transforming the latter into a combination optimization problem and using a nonlinear mathematical model for that purpose. Yang studied the effect of planting scheme on yield in intercropped high yield fields [9]. It turned out that the main factors to determine corn yield under intercropping were breadth of stripes and plant height. So far, little attention has been paid to the interspecific dynamics and temporal stability of intercropping productivity: Dong found that the majority of intercropping systems not only exhibited efficient resource utilization and a yield advantage, but also sustained spatio-temporal stability of productivity and nutrient uptake [10]. Karthikeyan and Jawaharlal conducted an experiment on optimization of plant density inside a greenhouse for growing carnation [11]. They found that at a planting density of 20 plants/m^2^ a better percentage of ‘A’ grade quality flowers was obtained, whereas a density of 36 plants/m^2^ proved to be better in terms of flower quality parameters, such as early flower bud appearance, bud opening, longer duration of flowering, chlorophyll content and increased number of flowers per plant, all of them contributing to a higher economic value of the crop. Jiang used the DSSAT crop growth model to optimize and screen for the best plant model within crop cultivation schemes [12]. Grahmann *et al*. used conservation agriculture to optimize nitrogen management in an agricultural system based on minimum tillage, crop residue retention and crop rotation [13], Dai *et al*. used a modern design optimization method to simplify parameters in the process of transplanting mechanism and used mathematical modelling to analysis the kinematic constraints of the transplanting mechanism [14].

The above-mentioned studies were mainly experimental (i.e. carried out under field conditions) whereas only a few studies with virtual plants have been conducted due to the limitations of plant modelling. In other words, a plant model should consider proper aspects to integrate impacts both from within the plant, e.g. major physiological processes, and from its environment, e.g. radiation and temperature, while most previous models have focused on only few factors, which render them less suitable for their being integrated in other applications. Fortunately, with the progress in modelling research, recent plant models (notably functional-structural plant models, Vos *et al*. [15]) now include an appreciable number of modules describing basic physiological processes [16–19] and are therefore more comprehensive and realistic than previous model generations. Therefore, an adapted optimization algorithm was introduced in the present study, to be integrated with the virtual rice model, and to obtain optimal spacing for rice individuals under certain climate settings.

The rice model was chosen because of its consideration of plant architecture and its dynamics in time, and extended to consider information about organ dynamics (number, size, and mass) and the position and orientation of plant parts in 3-D space during development. Furthermore, with the implementation of a properly calibrated rice model, not only can the 3-D representation of the data sets be obtained, but one can also envisage the possibility to predict its formation [2].

In light of the knowledge gaps described above, it was the objective of the present study to use a virtual plant approach in combination with the newly developed Mixed Particle Swarm Optimization algorithm, to obtain a rapid workflow for optimal spacing of crop plants with respect to light interception. Our hypothesis is that our virtual plant approach is sufficient to predict basic crop behaviour in terms of agronomic output (biomass production and carbon allocation, leaf surface), because light interception is by far the most important physiological factor.

## Materials and methods

### Virtual rice model

A previously developed Functional-Structural model of rice [2], implemented in the modelling platform GroIMP [20] was used in this study. The rice model integrates major physiological functions together with processes describing vegetative and generative morphology in a simplified way. Moreover, a separate parameter module contains the general settings of the system and the environmental parameters. The data and model code, plus instructions to run the model, can be found in this GitHub repository: https://github.com/lfxu1/riceSpacingOpt.

**Fig 1:**
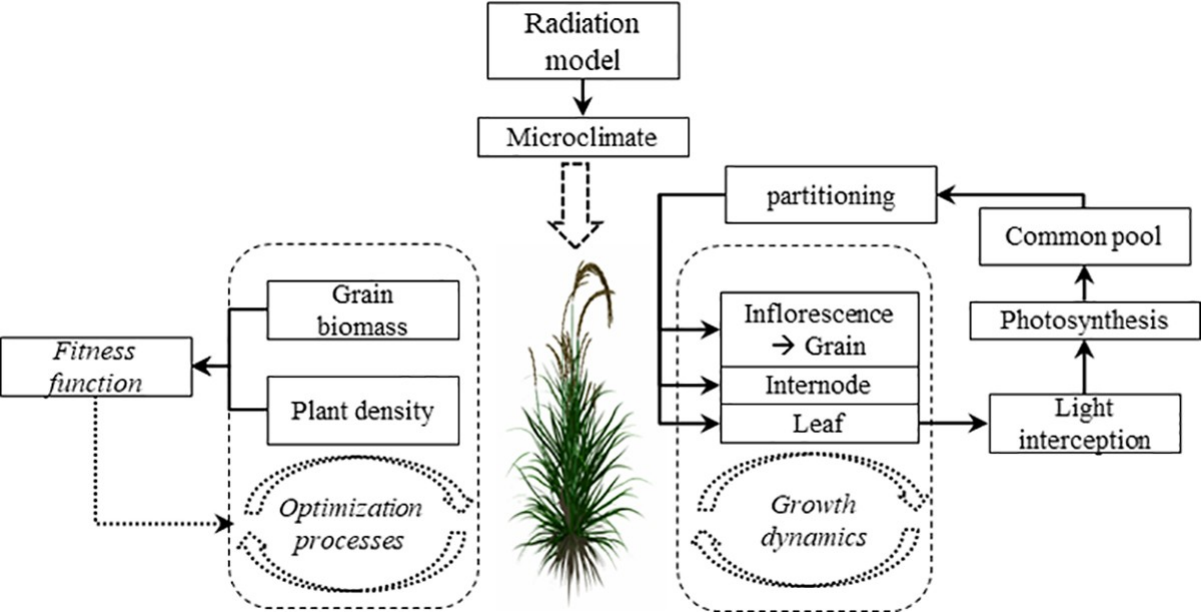
Diagram illustrating the main processes of the functional-structural rice model and interaction with the MPSO algorithm.

The environment parameters (PAR, temperature, day length) are mainly used for the calculation of light interception by the modelled leaves and for the corresponding biomass produced by photosynthesis. The processes involving the amount of biomass as state variable can be subdivided into three parts: growth (including growth respiration), maintenance respiration, and biomass distribution. Maintenance respiration represents a simplified quantification of glycolysis to produce metabolic energy units used for life-sustaining processes, i.e. the maintenance of the living biomass already produced, whereas growth and branching describe the dynamics of the formation and elongation or weight gain of new structures, leading to canopy architecture, developmental dynamics and biomass dynamics. The architecture of the plant will have an influence on the change of light interception with environmental parameters. In summary, these processes describe the connection of different modules within the plant model, and reproduce the main features of growth and development of the actual plant (as shown in Fig 1).

### Fitness of optimization

Plant growth is driven by several factors: the number and sink strength of growing organs will determine the main patterns of biomass accumulation and dry matter partitioning within the plant. Photosynthesis is the central process in the source organs (leaves), providing sugars for respiration and growth. Light interception, representing the main process leading to energy supply for photosynthesis, has a great influence on growth, and vice versa: High population density, coupled with high branching intensity or not, will have an effect on light microclimate, possibly allowing less light to reach the lower leaves, the latter leading to lower photosynthetic capacity. On the other hand, morphological changes (less branching and more internode elongation) are induced by a lowered R/FR ratio (thus light quality, not quantity), not by a depressed photosynthetic capacity [21]. Adjustment of population density can significantly change the growth dynamics of plants, and have further effects on final yield production. Photosynthetic production can be adjusted in such way. To simulate it, yield per unit area was used as the fitness function (Eq (1)):

fitness=biomass/area (1)

where *biomass* denotes the sum of each individual’s grain biomass, as shown in Eq (2); *area* denotes the plant population’s soil surface.

biomass=f(environmentalfactors,plantspacing,organs) (2)

With respect to the modelling of the rice plant, the environmental factors are also considered. Measured hourly and daily climate parameters for Hangzhou (P.R. China, latitude 30°16′ N, longitude 120°11′ E) [2] were used in this study. Competition among neighbouring plants for light and space was considered in the rice model. In this study, the basic growth procedure of virtual rice follows the same routine as in a previous study [2], while a new optimization strategy combined with MPSO was designed and combined with the rice model. Within the optimization algorithm, plant spacing was the only feature that MPSO adjusted in each iteration. Plant spacing was considered as a particle coordinate in MPSO.

### MPSO

Compared to PSO [22], the recently developed optimization algorithm MPSO can be used to calculate the optimal value in a relatively more efficient way, while at the same time the convergence precision of MPSO is significantly higher [23].

**Fig 2:**
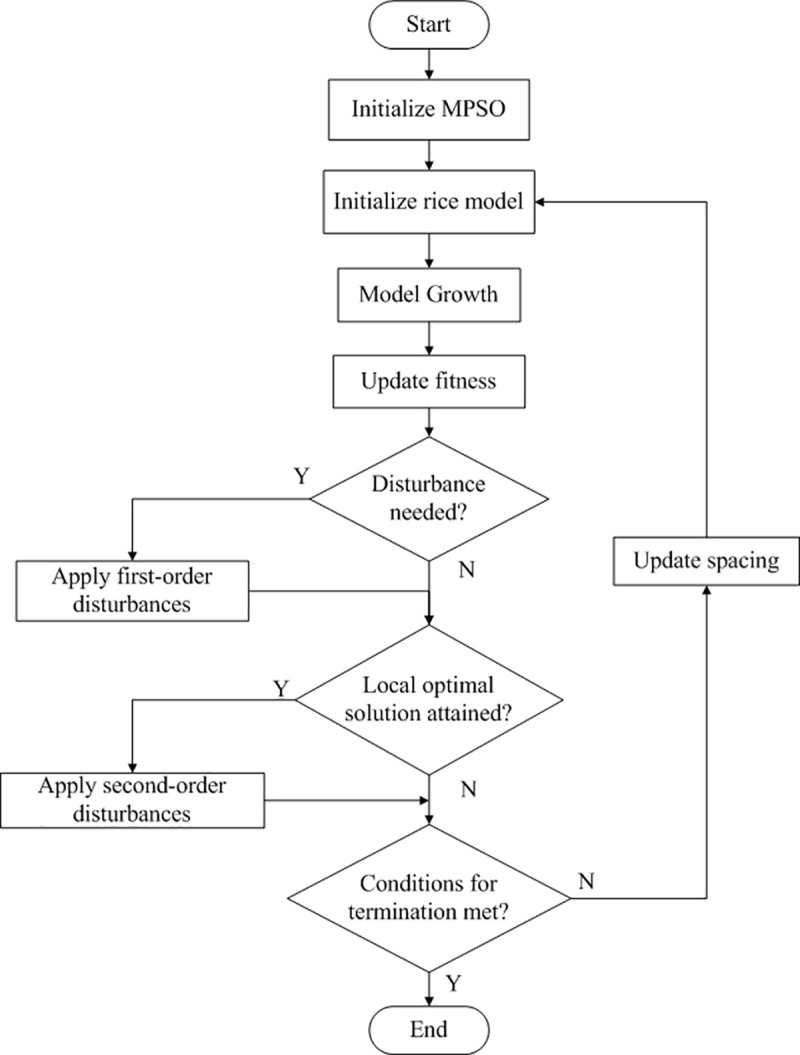
Diagram representing the flow chart of MPSO.

The optimization process of MPSO can be divided into two stages: explore and converge. At the early stage, the priority is on exploration, and extra disturbances are applied to improve exploration capability while avoiding local optima; at a later stage, the algorithm tends to converge, and weak disturbances are applied to this convergence. It was proven that MPSO performed better with multimodal functions [24]. A flow chart depicting the principle of MPSO is shown in Fig 2.

The workflow of combining the MPSO with the rice model is as follows:

Step 1. Initialize parameters of MPSO, e.g. maximal optimization time (i.e. the point when the algorithm stops optimization), number of particles, time range for changing the update function and parameters of the update function.

Step 2. Initialize model settings, e.g. initial spacing, population size, environmental parameters; reset the growing situation (the environment and growing conditions) and reinitialize the growth stage back to seedling.

Step 3. Run rice model with individual growth dynamics until max growth time is attained.

Step 4. Calculate total grain biomass for simulated rice plants.

Step 5. Use grain biomass with soil surface area to compute current fitness, then use a new equation (Eq (3)) to determine whether to introduce the disturbance; if yes, introduce first-order disturbance; if no, go to Step 6;

Step 6. Count number of times the fitness value stays the same; if this number is 10 or more this means a local optimum solution was obtained: introduce second-order disturbance; if not, go to Step 7;

Step 7. Decide if termination conditions are met: if yes, end optimization; otherwise, update spacing and return to Step 2.

The update functions of Step 7 are shown in Eq (4); the first-order disturbance function in Step 5 is shown in Eq (5A); the second-order disturbance function in Step 5 is shown in Eq (5B);

$$pr=\cos(\frac{\pi *cg}{2*m_{T}})/2$$ (3)

$$\begin{array}{l} V_{i}^{k+1}=\{\begin{array}{l} \omega V_{i}^{k}+c_{1}r_{1}(P_{i}^{k}-X_{i}^{k})+c_{2}r_{2}(P_{g}^{k}-X_{i}^{k}),\;(a)\\ \phi (V_{i}^{k}+c_{1}r_{1}(P_{i}^{k}-X_{i}^{k})+c_{2}r_{2}(P_{g}^{k}-X_{i}^{k})),\;(b) \end{array}\\ X_{i}^{k+1}=X_{i}^{k}+V_{i}^{k+1} \end{array}$$ (4)

where *V*_*i*_^*k*^ denotes the velocity of *k*th iterative depth and *i*th particle. *X*_*i*_^*k*^ denotes the spacing of *k*th iterative depth and *i*th particle, *ω* denotes inertia weight, *φ* denotes a constriction factor, *c*_*1*_ and *c*_*2*_ denote learning factors: they represent the ability to inherit from the previous velocity of a particle; *r*_*1*_ and *r*_*2*_ are random numbers in [0, 1], whose values will be discussed in the Results. Function (a) is the update function before the time changing point, function (b) the update function after the time changing point,

$$\begin{array}{l} A:\{\begin{array}{c} X_{i}^{k}=r_{0}*X_{i}^{k}\\ X_{i}^{k}=(r_{1}+r_{2})*r_{3}*X_{i}^{k}\\ X_{i}^{k}=ga*X_{i}^{k}\\ reset \end{array}\;k<T\\ B:\{\begin{array}{c} X_{i}^{k}=r_{1}*X_{i}^{k}\\ X_{i}^{k}=(r_{1}+r_{2})/2*X_{i}^{k} \end{array}\;T\leq k<T_{m} \end{array}$$ (5)

where *r*_*1*_,*r*_*2*_,*r*_*3*_ are random numbers in [0,1], *ga* the Gaussian random number; *reset* denotes the resetting of the *X*_*i*_^*k*^ value to a new random spacing within the solution domain; *T* denotes the time changing point; *T*_*m*_ denotes the maximum time of optimization.

$$X_{i}^{k}=(X_{gb}^{k-1}+X_{gb}^{k+1})*r_{a}$$ (6)

where *r*_*a*_ is a floating point random number between [–2, 2], *X*_*ga*_^*k-1*^ and *X*_*ga*_^*k+1*^, respectively, indicate the optimal position values of the current population of the *k-1* th and *k+1* th generations.

## Results

For the simulation calculations, a computer with the following hardware configuration was used: i3-6100 CPU, 4GB RAM.

The parameters used for the MPSO are shown in Table 1.

**Table 1:** Parameters used within MPSO.

Parameters	*Value/Range*
Optimization times	300
Population of swarm	5
*c*_*1*_ (Pre-learning)	2
*c*_*2*_ (Pre-learning)	2
*φ* (inertia weight)	[0.9, 0.4] (as Eq (7)) [18]
*c*_*1*_ (late learning)	2.05
*c*_*2*_ (late learning)	2.05
*γ* (contraction factor)	0.729 [22]
Number of repetitions	5

$$\omega =0.9-0.5*\log_{T_{m}}T_{c}$$ (7)

where *T*_*m*_ denotes the maximum optimization time, *T*_*c*_ denotes the current time.

The population size of the virtual rice in the model was set to 36 individuals (6 plants x 6 rows). The grain biomass from the inner 4 by 4 plants as well as the corresponding ground floor area were considered in the optimization workflow (border plants were discarded as they presented a boundary effect, notably a surplus of laterally intercepted light).

The results are likely to be different under various settings for the time of switching point (from the period of exploration to conversion) during each optimization procedure. To get the optimal range, some extra optimization experiments were performed to test the behaviour for each setting of the switching point. Based on the results of those experiments (the switching point results are shown in Table 2), the switching point range was set as [75, 225].

**Table 2:** List of switching points in optimization procedure.

Number	*Switching point in optimization (max time*: *300)*
1	175
2	203
3	111
4	83
5	81

**Fig 3:**
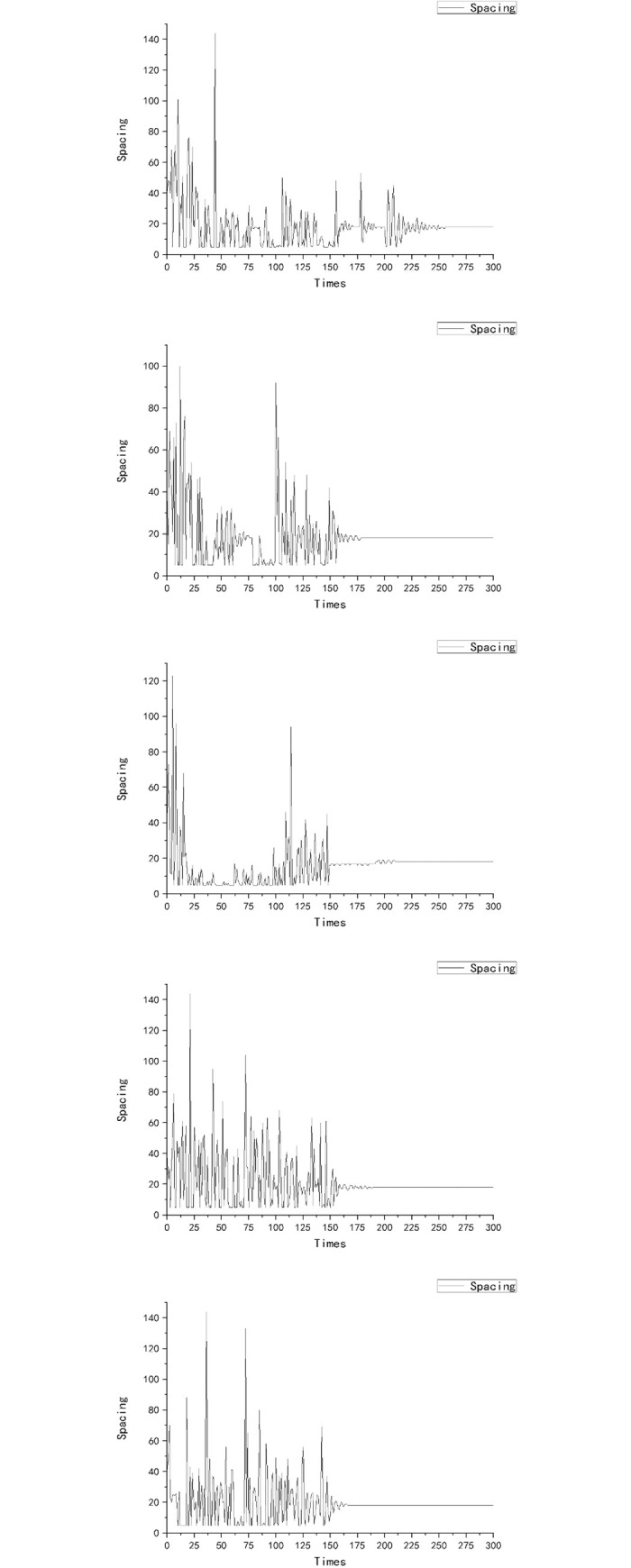
Dynamics of rice spacing within the optimization procedure with MPSO applied on a 3D rice model.

**Fig 4:**
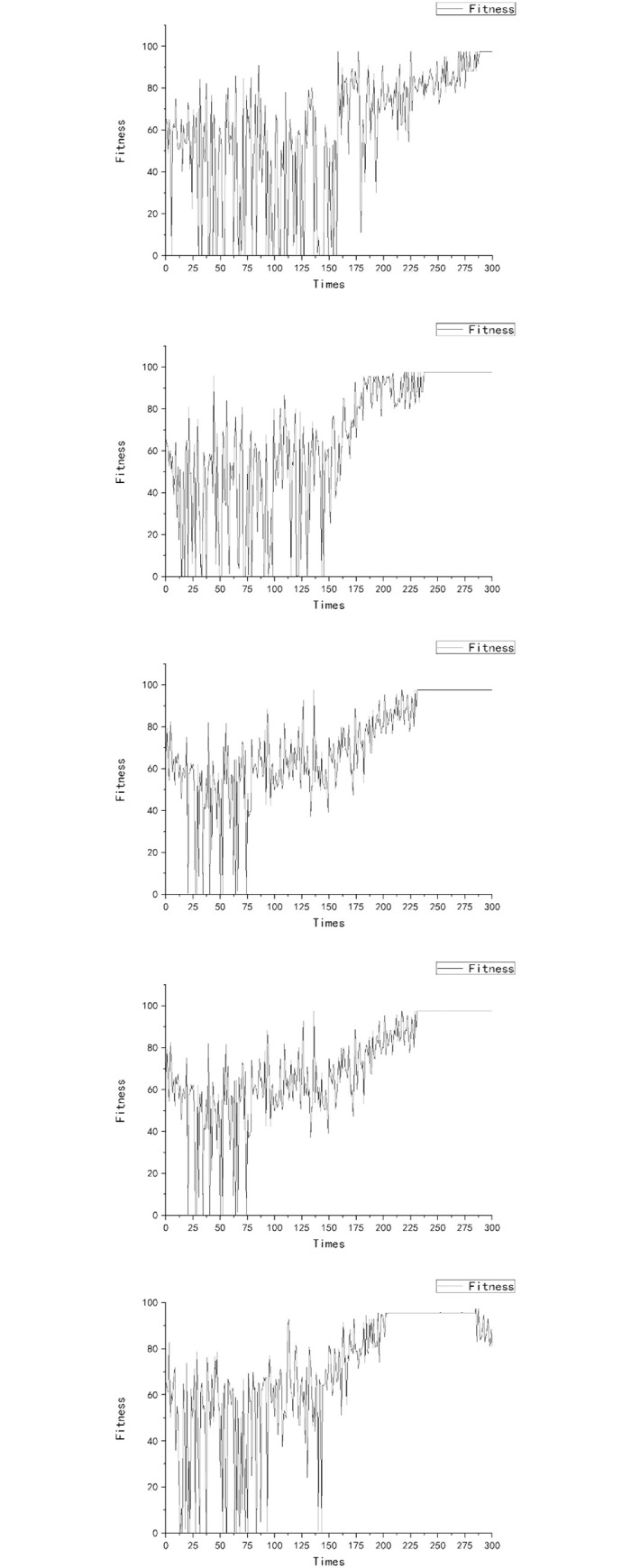
Dynamics of fitness within the optimization procedures with MPSO applied on a 3D rice model.

**Fig 5:**
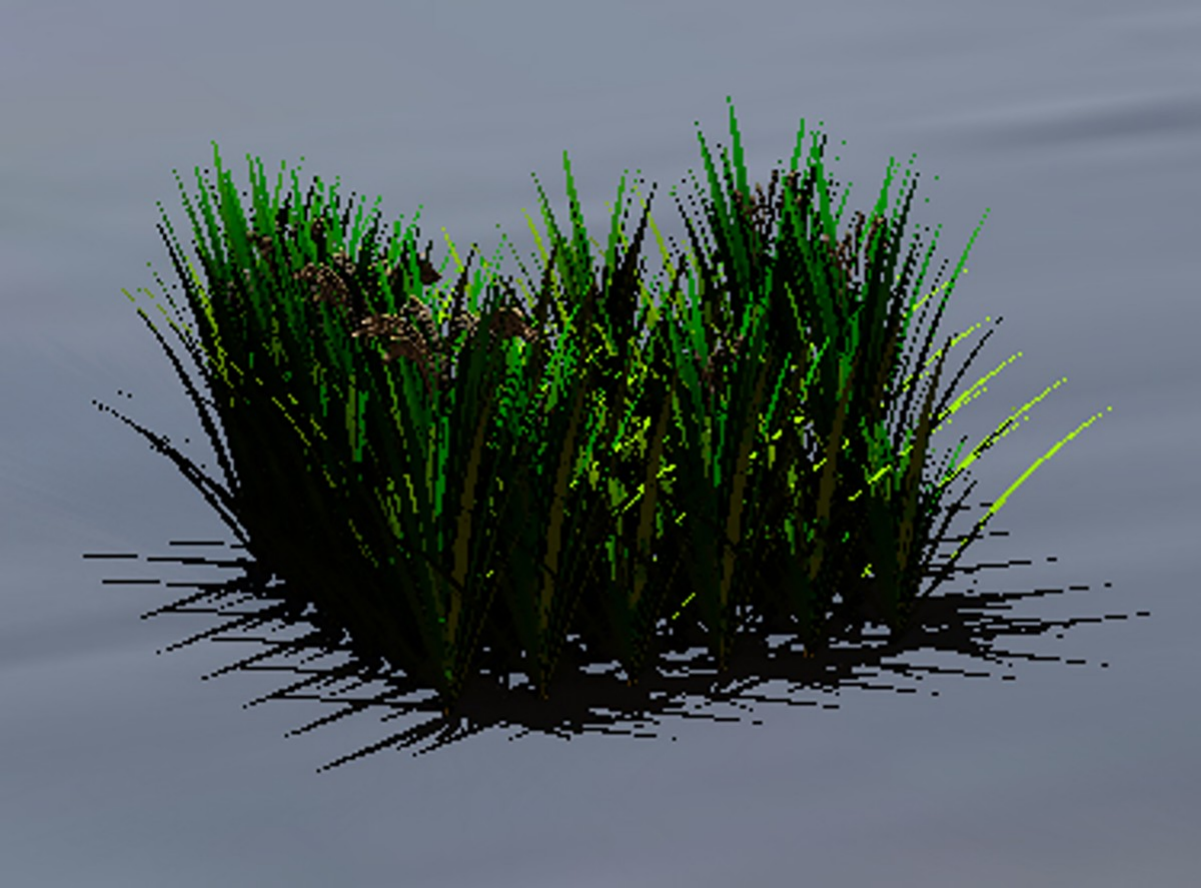
Final growth status of simulated rice plants (spacing: 18.3 cm) with different phenotypes. A population of 6 x 6 individuals is depicted.

**Fig 6:**
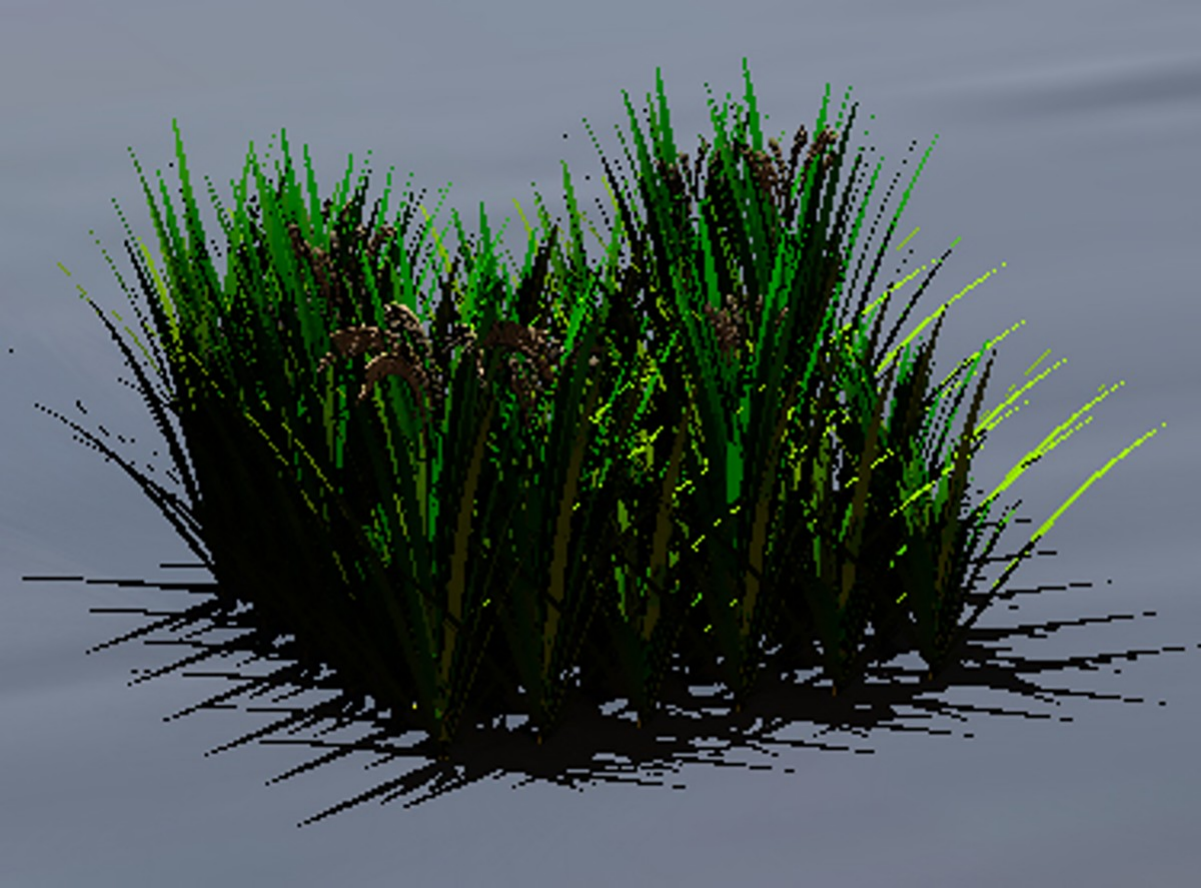
Final growth status of simulated rice plants with spacing of 18.5 cm.

Optimization results for rice spacing are shown in Table 3. Fig 3 shows the dynamics of spacing during the process of optimization, while Fig 4 shows the change of fitness value under the process of optimization. The final stage of growth of the rice population with the specified spatial settings is shown in Figs 5 and 6.

**Table 3:** Optimal spacing and corresponding fitness value for the five optimization experiments based on the rice model.

Number	*Best Spacing(cm)*	*Best Fitness (biomass(mol)/area(cm*^*2*^*))*
1	18.3	97.50
2	18.3	97.50
3	18.5	95.69
4	18.3	97.50
5	18.3	97.50

Fig 3 shows the relation between iteration depth and plant spacing. It can be observed that at the early iteration stage (depth from 0 to 150), the algorithm performed well in searching and was able to get rid of the local optimal values; while at the late iteration stage (depth from 150 onwards), the algorithm had a relatively better performance in conversion. Fig 4 shows the relation of iteration depth and fitness value: at the early iteration depth, the fitness value changed all the time, while at the later iteration depth the fitness value was stable with a maximum value (except experiment E). Figs 3 and 4 show that at the late iteration depth the plant spacing of experiment E did not change, but the fitness value was changed at the final iteration depth. This may be due to the randomness of the model during dynamic growth. In general, while even the optimized algorithm in the rice model exhibited some randomness, the repeated optimization results showed a certain stability of both the rice model and the optimization algorithm. In five experiments, the best plant spacing values were found at around 18 cm. This showed the stability and reliability of these experiments. Furthermore, the process of optimization experiments showed that the optimization algorithm had superior spatial exploration and convergence performance. With respect to the fitness function, it can be concluded that the highest biomass per unit area under such model and environmental settings was obtained with the rice model when the spacing of the virtual plant individuals was 18 cm (inter-row and inter-plant).

## Discussion

Using an integration of a functional-structural rice model and the MPSO algorithm, optimum plant spacing with respect to grain yield per ground surface was obtained. Within the MPSO, biomass per unit area was chosen as the fitness function, i.e. a criterion within the algorithm to evaluate the simulation results. Compared to the amount of light intercepted, which also could have been used as the fitness function and which is seemingly more directly related to plant spacing, biomass per unit area is a factor that exhibits a stronger positive correlation to final yield. The average yield for rice individuals per unit area is not necessarily the largest when each individual intercepts a maximum amount of light throughout the growth period. Moreover, biomass per unit area results from light interception together with the planting area, which is a more representative measure for total crop yield per ground area. There are other studies combing optimization algorithms with growth models [25,26]. The focus in this study was to optimize plant spacing for rice individuals within a functional-structural rice model.

The simulation results from this study indicated that the optimized spacing of virtual rice plants was 18 cm. Though we did not validate our model with an independent dataset, a comparison with the recent literature shows that this result is within the range of optimal row spacing suggested by other studies, i.e. 15–20 cm of row spacing was recommended to produce maximum yields by Hardke et al. [27], and 18–27 cm by Dunn et al [28]. It is understood that this result was obtained under a number of simplifications, e.g. fixed number of tillers and fixed distribution of plant height to internode length. In our 3D model, leaves dynamically unfold during development, but we have not implemented a feedback between final leaf angle and exposure of the leaf blade to local light availability. Another simplification of our model is the use of a central carbon pool. The daily assimilate production is transferred to this central reservoir, and then assimilated carbon is redistributed to each organ according to the growth and respiration requirements of each organ (relative sink strength principle) [29]. This implies that carbon remobilization from the flag leaves to developing grains is presently neglected. In addition, our model did only explore regular drill sowing: for other arrangements like broadcasting or quincunx sowing, different optimal distances and overall densities might have been found [30]. The present study only represents the first steps towards an optimization method of planting density with FSPMs on rice cultivars. Plant height was fixed, too, for each individual within the population during the optimization procedure. It is true that different canopy heights may greatly change the intermediate values within the optimization algorithm, and the above-mentioned results may not be valid for new settings. Besides, light plays an important role in the regulation of tillering, and the ratio between the intensities of red and far-red light (R:FR) has been linked to tillering in Poaceae [31]. In this study, a basic rice FSPM was combined with a new optimization algorithm, MPSO, and a framework for the optimization of the spatial distribution of rice was proposed. The next step would be to adjust the optimization workflow to consider a virtual rice canopy with different heights and plant types. In future studies, intercropping models of different crops (e.g. maize/soybean) will be tested using the same optimization framework.

It is efficient and useful to combine plant models instead of actual crop experiments in an optimization study of plant spacing. Using models in agronomic planning and research could on the one hand save time and human resources; of course, on the other hand, the ability to simulate faithfully growth dynamics with plant models is a huge challenge. Field experiments would almost fully reflect the performance of a plant during all growth stages, while it would also come with certain risks and disadvantages, e.g. unfavourable climate, pests, as well as the problem associated with the complexity and tediousness of phenotypic data acquisition (measurement errors and operator bias). A plant model was used in this study to substitute the actual crop: this rendered data acquisition, observation and maintenance unnecessary, respectively, replaced data acquisition by simulation, which ensured the stability and robustness of the plant model in the optimization processes. Integrated with the extended optimization algorithm, the validity of results was further improved. Combining a plant model with an optimization algorithm may be useful to guide agricultural production in the future. However, there is still considerable room for improvement with respect to the combination of the virtual model and the optimization algorithm, such as the setting of target fitness. In this study, we used the grain biomass per unit area as the target fitness of the rice growth. However, this fitness function only considered the grain of rice, excluding other aspects of the developmental physiology, which means the modelled rice considers potential production conditions (no biotic or abiotic stress). This is, of course, impossible in agricultural production, so in the next version of the model we will modify the target fitness function to make it include abiotic and biotic stress (climate and pests). This will add validity to the fitness function due to the robustness in MPSO.

## Conclusion

In the present study, MPSO was used to optimize plant spacing in a 3D rice model. It served as an external tool with the objective of increasing the total grain biomass by adjusting the spacing of rice individuals. Growth behaviour of virtual rice was mainly controlled by rules for vegetative and generative growth in a mechanistic way. At the same time, the different row and interplant spacing values of rice individuals during the optimization process affected the light microclimate around the leaves, thus light interception of the individuals was influenced, leading to more light interception at larger plant spacing within a certain range, and vice versa. However, the increase in intercepted light will stop at a certain value due to the limited leaf area. MPSO helps to find the optimal distance settings for the rice model considering intrinsic (physiological) and environmental conditions. Finally yet importantly, the final growth status of simulated rice used grain biomass per unit area as a target fitness function to optimize plant spacing.
